# Tourism management in national parks: Šumava and Bayerischer Wald (Bavarian Forest) in the Czech-German borderland

**DOI:** 10.1007/s11629-021-6853-9

**Published:** 2021-09-20

**Authors:** Krzysztof Kołodziejczyk

**Affiliations:** Department of Regional Geography and Tourism, Institute of Geography and Regional Development, Faculty of Earth Sciences and Environmental Management, University of Wroclaw, 54-152 Wroclaw, Poland

**Keywords:** National parks, Tourism management, Tourism infrastructure, Tourist trails, Šumava, Bayerischer Wald (Bavarian Forest)

## Abstract

Along the Czech-German border there are four national parks, two Czech and two German, arranged in cross-border ‘pairs’. This article focuses on the southern ‘pair’ formed by the parks of Šumava and Bayerischer Wald (Bavarian Forest). The aim is to evaluate and compare tourism organization in their areas, taking into account selected aspects of management: the network of hiking trails with its related infrastructure, transport accessibility, a typology of tourist centers, as well as directions and destinations of tourist movements. Based on the results, it can be concluded that the availability of geographical space for tourists is much greater in the German than in the Czech national park, and the tourism infrastructure is clearly more extensive there, including the network of tourist trails. This is mainly due to the longer and fairly uninterrupted development of tourism in this area. Šumava National Park can be identified as a model in terms of how to adjust the directions of tourist movements and the layout of the tourist trail network to the needs of natural environment. On the basis of observations in both national parks, it is possible to indicate various solutions that, after appropriate adaptation, may bring benefits to other protected areas.

## 1 Introduction

Along the Czech-German border there are four national parks, forming two cross-border 'pairs', one to the north, the other to the south (Fig. 1). The northern pair comprises the Bohemian 'Switzerland' National Park (Czech: *Národní park České Svýcarsko*) and the Saxon 'Switzerland' National Park (German: *Nazionalpark Sächsische Schweiz*), while the southern – the Šumava National Park (Czech: *Národní park Šumava*) and the Bavarian Forest National Park (German: *Nazionalpark Bayerischer Wald*). The characteristic that links these areas is the relief of 'medium-scale' mountains, however, each 'pair' represents a different kind of geographical environment. For the northern pair sandstone landscapes are characteristic, moreover, the area is cut by an impressive gorge of the Elbe. In the southern pair we deal with geologically old mountains with some Pleistocene glacial relics (cf. Balatka and Kalvoda 2006; Czudek ed. 1972; Härtel et al. 2007; Pánek and Hradecký 2016). This situation enables various comparative analyses. As national parks in many locations are places of intensive tourism development, a tourism management perspective should be incorporated in these studies, including the level of accessibility to tourists of the protected space on each side of the border or the development of tourist facilities. It is also advisable to consider the potential consequences of decisions concerning tourism management for the neighboring protected

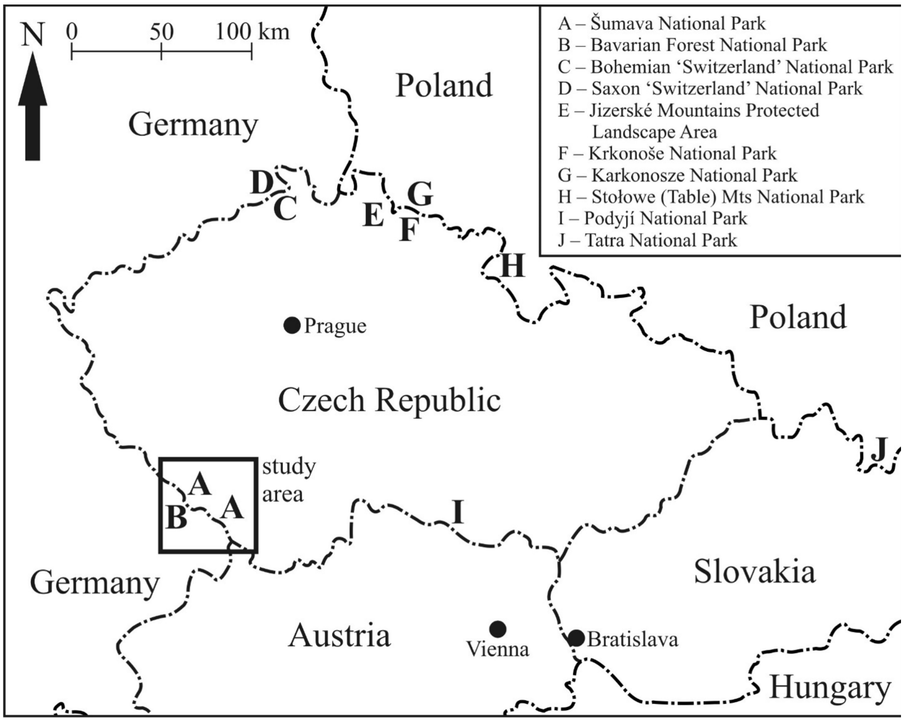

**Fig. 1** Location of the national parks analyzed and other protected areas widely mentioned in the article.

area. As a continuation of the work on Bohemian and Saxon 'Switzerland' (Kołodziejczyk 2020d), these issues will be analyzed based on the example of Šumava and Bayerischer Wald National Parks.

The organization of tourism in national parks, or more broadly in legally protected areas, is a multiple issue (Ceballos-Lascuráin 1996; Dudek 2017; Eagles 2008, 2009; Islam et al. 2018; Kolasińska 2010; Leung and Marion 2000; Manning et al. 2017; Moyle 2013; Ptaszycka-Jackowska and Baranowska-Janota 1989, 1996; Tomczyk and Ewertowski 2013) covering natural conditions (including terrain relief, the river network together with wetlands, flora and fauna, and the distribution of particularly valuable areas where human interference should be maximally limited), social conditions (demand for tourism and recreation, popularity of individual destinations, predominant models of tourism) and infrastructure (transport accessibility, existing and planned tourism infrastructure). This article focuses on the last aspect, but includes natural conditions as well. Tourism management in protected areas should follow the rules of sustainable development (Buckley 2012b; Larson and Poudyal 2012; Leung et al. 2018). Topics usually analyzed in this context are i.a.: positive and negative impacts of tourism, both on natural environment and society or culture (Buckley 2004, 2010; Chandralal 2010; Wall and Mathieson 2006), monitoring of tourist flow and the changes caused (Gitzen et al. 2012; Miller and Twining-Ward 2005), carrying capacity (Cole 2004), and management of revenues and costs (Eagles 2002; Eagles and McCool 2002; Lepp 2007). Legal and organizational conditions of individual forms of nature protection should be taken into account, as well as the natural and social features of specific areas.

Apart from accommodation and catering facilities (which should be concentrated in the surroundings of a protected area and not within its borders), the key role in tourism infrastructure in national parks is played by tourist trails. According to most legal regulations, they are often the only way to travel around a national park (or at least selected parts, most often referred to as the core or strict protection zone). According to many authors (Kolasińska et al. 2015; Liszewski 2009; Ptaszycka-Jackowska and Baranowska-Janota 1989, 1996; Styperek 2001, 2002b), tourist trails are one of the most important elements in the management of national parks. Organization of tourist movements within their areas, including both individual and group penetration, should result from variants of visitor programs, constructed through the shape of the trail network, its capacity and the monitoring of the actual number of tourists visiting the entire park, its individual parts, as well as its surroundings. Tourist trails are an important element of management in national parks as they facilitate basic functions of such protected areas: nature conservation, making the area available to tourists, specialized education and scientific research. At the same time, tourist trails in national parks themselves fulfil a number of functions (Stasiak 2006, 2007; Styperek 2002b). The first one, connected directly with tourism, is to make the tourism value of the area accessible, to enable tourists to see and appreciate local attractions and other assets of the natural environment. The second one (ecological) is to channel tourists along certain zones, allowing penetration to be limited or even excluded in particularly valuable areas or locations with a low resistance to anthropopression (Getz 1983; Kolasińska et al. 2015; Marion and Leung 2004). In more detail, Styperek (2002b) distinguishes the following as part of the tourism function of trails: sightseeing (making the value of the natural environment, cultural assets and the products of contemporary human activity accessible), recreational (regeneration, management of free time, relaxation through contact with nature, satisfying 'higher' human needs) and sport (enabling cross-country running, climbing, cycling, skiing, canoeing, etc.). On the other hand, within the group of ecological functions, Styperek (2002b) indicates a protective one (preventing degradation, excluding selected protected areas from tourist impact), 'steering' (stimulating the directions of tourist movements, controlling their intensity) and educational (shaping types of tourist and recreational behavior and social attitudes, enriching knowledge).

According to some authors (e.g. Stasiak 1997) the aim of tourism management in national parks should be to find a 'golden mean' between tourists' expectations and nature conservation regulations (cf. López and Pardo 2018). Solutions need to be found to give tourists access to attractions located within a protected area and at the same time to protect the natural environment from the negative impact these tourists may have, as far as possible. However, quite often expectations of tourist or tourist sector may be beyond the ecological or even tourism carrying capacity in a national park, which can lead to a conflict between users' prospects and the management goals of parks' authorities (Karhu et al. 2020), examples of which are various protected areas located not only on the border – e.g. the aforementioned Bohemian and Saxon 'Switzerland', the Tatra or Harz Mountains or Koli National Park. In the same time it has to be remembered that one of the main goals of recreational activities in national parks is education and interpretation and not every type of tourist or recreational action can take place in such areas. According to IUCN one of the objectives of a national park is "to manage visitor use for inspirational, educational, cultural and recreational purposes at a level which will not cause significant biological or ecological degradation to the natural resources" (IUCN n.d.).

## 2 Aims and Methods

The aim of the paper is to assess and compare the management of tourists in the Šumava (Czech Republic) and Bayerischer Wald (Germany) National Parks. The comparison will be made of solutions applied in each of the national parks, as well as attention will be paid to initiatives and activities functioning on both sides of the border simultaneously. In many cases the observations will be related to the second 'pair' of Czech-German border national parks as well as to other protected areas in Central Europe. The intention is to indicate solutions worth promoting and replicating (after necessary adaptation) in other borderland protected areas of a similar geographical environment. Taking into account the experience and practice in several parks analyzed, conclusions useful in other mountain national parks can be drawn, including more detailed, technical solutions. It is hypothesized that the historical conditions of the general development of the studied areas were of key importance for the development of tourism, especially the differences on both sides of the border. As a consequence, it is assumed that the level of tourist infrastructure development will be higher on the German side.

Selected aspects of tourism management in national parks were analyzed: the network of hiking trails with related tourism infrastructure, transport accessibility, a typology of tourist centers, directions and destinations of tourist movements. The choice of these aspects, which constitute only a part of all the issues connected with tourist management, was dictated by the fact that they can largely be shaped by national park authorities and local governments, contrary to accommodation and catering facilities, for example, usually privately funded, whose decisions are largely based on the criterion of profitability or the general economic situation. Tourism management in protected areas from a technical point of view often focus on capacity (Eagles and McCool 2002; Leung et al. 2018). Without underestimating it, in this article it was decided to focus on the ways of shaping the network of tourist trails and the directions of movement of tourists. The density and layout of trails affect the accessibility of individual areas and by adjusting them to the resilience of the environment, very positive results in the sustainable development of protected areas can be obtained.

As tourist trails play a key role in the organization of tourist movements within national parks, it is the development of their network that has received the most attention. The degree of development and coherence of the network of hiking trails in both protected areas has been analyzed. The choice of hiking trails results from three factors: 1) hiking is the most frequently undertaken form of recreational penetration (Sievanen and Knopp 1992; Sołtysik and Toczek-Werner 2010; Styperek 2002b; Toczek-Werner 2004; Vystoupil and Sauer 2012, 2017); 2) hiking trails are the only type of tourist trail common in all national parks in Central Europe, which makes comparisons possible; 3) hiking is subject to the least restrictions; 4) the development of hiking trails in national parks usually fully depends on the park authorities. The analysis of the hiking trail network was conducted using selected methods from graph theory, namely indices of graph's spatial structure. The network in both national parks was simplified to a planar undirected graph. As a consequence, it was assumed that each trail junction is a vertex on a graph, and the sections between the junctions are the graph's edges. In a situation when more than one official trail run the same way between neighboring junctions, only one edge is included. In this way, a schematic picture of the network is obtained, which, thanks to the removal of the winding nature of the actual routes, facilitates analysis and comparison. Referring to works by Kołodziejczyk (2014b, 2015a, 2018, 2019, 2020c), Krakowiak (1997), Styperek (2001, 2002a, 2002b), and Taczanowska et al. (2014, 2017), the following indices were used (Taaffe and Gauthier 1973; Potrykowski and Taylor 1982; cf. Gross et al. 2018):

(1) Kansky $\beta$ index = $e/v$, where $e$ is the number of edges, $v$ is the number of vertices (the higher the value of this index, the more coherent the network of trails),

(2) Kansky $\gamma$ index = $e/[3\times(v-2)]$ (the ratio of the number of existing edges to the maximum possible number of edges resulting from the number of existing vertices; the values of the Kansky $\gamma$ index range from 0 for total incoherence to 1 when all possible edges are present),

(3) $\alpha$ index = $\mu/(2v-5)$, where $\mu$ is a cyclomatic number calculated based on the equation $\mu = e - v + p$, where $p$ is the number of isolated subgraphs.

According to the values of the $\alpha$ and $\gamma$ indices it is possible to assign a given network of trails to a certain type of network shape. The categorization used in the article was developed by Taaffe and Gauthier (1973) and later modified by Styperek (2001). They singled out (Table 1): 1) the core scheme (least developed and coherent, practically only a main route with access trails), 2) the core-lattice scheme (slightly more developed, with a higher proportion between edges and vertices), 3) the lattice scheme (with even more connections) and 4) the delta scheme (the most coherent one, with all possible connections between junctions, actually really rarely existing). As an additional aspect, all tourist facilities functionally

**Table 1** Classification of network shapes depending on the values of topological analysis indices sourced from graph theory

| Core scheme | Lattice scheme | Delta scheme |
|---|---|---|
|  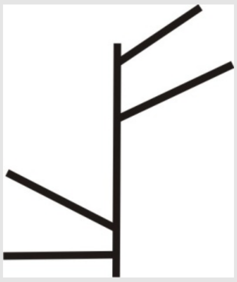 |  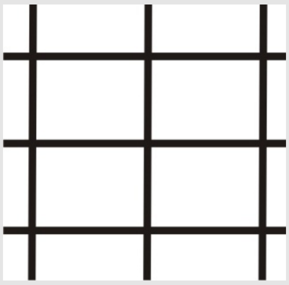 |  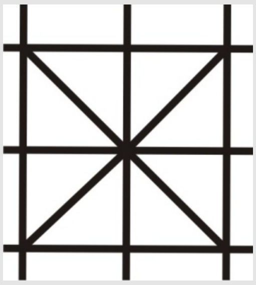 |
| $1/3 \le \gamma \le 1/2$; $v \ge 4$<br>$\alpha = 0$ | $1/2 \le \gamma \le 2/3$; $v \ge 4$<br>$0 < \alpha < 1/2$; $v \ge 3$ | $2/3 \le \gamma \le 1$; $v \ge 3$<br>$1/2 < \alpha < 1$; $v \ge 3$ |

**Note:** According to Styperek (2001), the characteristics of the mixed, core-lattice scheme are: $1/3 \le \gamma \le 1/2$; $v \ge 4$; $0 < \alpha < 1/2$; $v \ge 3$.

Source: Taaffe and Gauthier (1973), modified by Styperek (2001).

connected with hiking trails were investigated. Moreover, as the study covers national parks located on both sides of the state border, the number and distribution of places where this border can be crossed within protected areas using marked tourist trails were also analyzed (Kołodziejczyk 2020a; cf. Dołzbłasz 2017; Więckowski 2010). The work was based on an analysis of paper tourist maps (including: Geodézie on Line 2014, 2018; SHOCart 2002; update based on interactive tourist maps available on the internet, especially: Seznam n.d.) and field research carried out in 2005 (Šumava) and 2019 (Šumava, Bayerischer Wald).

Transport accessibility is also important for tourism management (Celata 2007) because it determines the possibilities of tourists' movement in the region. This issue can be analyzed from two perspectives (Kowalczyk and Derek 2010): 1) external accessibility determines the possibilities of travel from a tourist's place of residence to a tourist reception area; 2) internal accessibility determines the possibilities of movement within a reception area, in particular, reaching individual tourist attractions. The analysis focuses on public transport intended to facilitate tourist' needs, and the cross-border offer, i.e. the possibility of crossing the national border and visiting neighboring protected areas using regular bus routes and trains (see: Cavallaro and Dianin 2019; Kołodziejczyk 2020b). Available connections, departure and arrival times, and frequency were taken into account, also including seasonality of the offer. In the analysis of transport accessibility as of 2019, available timetables and connection search engines as well as field observations were used. Transport accessibility was determined on the basis of the number of connections available in individual parts of both national parks, including long-distance connections and those intended specifically for tourists (e.g. cyclobuses). Additionally, the adjustment of departure times to the tourists' needs was taken into account and the distribution of bus stops referring to the network of tourist trails.

The last element was to define the main directions of tourist movements and to classify tourist centers within both protected areas analyzed and their immediate vicinity regarding their functions. The directions of tourist penetration have been determined taking into account the course of roads, the public transport offer, the layout of the tourist trail network and random observations of the intensity of tourist movements in selected parts of national parks (traffic, public transport use and the numbers of tourists on chosen tourist trails were considered). The typology of tourist centers was made taking into account tourism infrastructure in certain settlements including accommodation and catering facilities, tourist information centers and sports facilities (Table 2). This part is a synthesis of previous analyses.

## 3 Study Area

Šumava together with the neighboring Bavarian Forest, located in the southern part of the Czech-German borderland (Fig. 1), are often referred to as the 'Green Roof of Europe' (Křenová and Kiener 2012; Meyer et al. 2009) as they comprise the most extensive forest complex in Central Europe (923 km$^2$). The area is also a UNESCO biosphere reserve, while

**Table 2** Typology of tourist centers serving borderland national parks

| Hierarchical level | Characteristics |
|---|---|
| Primary tourist hub | Main road and rail hub (including long-distance and international connections). Very well-developed accommodation (including hotels of various standards), catering and accompanying facilities. Tourist information center. An offer for both active and cultural tourists. |
| Secondary tourist hub | Local road and rail hub (few long-distance connections, possible local international border connections). Well-developed accommodation and catering facilities, basic accompanying facilities. Tourist information point. Offer targeted mainly at active tourists (junction of main tourist trails leading to the most important attractions). |
| Existing local tourist center | Average accessibility (usually only local bus routes). Quite well-developed accommodation and catering facilities. Basic sport infrastructure. Most commonly a tourist information point. A junction of tourist trails but of local importance. Both less and more popular attractions nearby. |
| Potential local tourist center | Weak accessibility. Few lower standard accommodation facilities. Lack of or scarce catering facilities. A junction of tourist trails but of local importance. Fairly attractive yet not popular surroundings. |

Source: Kołodziejczyk (2020d).

the local peatbogs are protected by the Ramsar Convention for the Protection of Wetlands (Kušová and Těšitel 2016; Těšitel and Kušová 2010). Located in southwest Czech Republic, the Šumava National Park was established in 1991, soon after the political and economic transformation. It covers an area of 680.64 km², being the biggest of four national parks in the country. An important characteristic of the park from the spatial point of view is that it stretches from northwest to southeast along the national border (Dickie et al. 2014) – in the north-western and central parts with Germany, and in the south-eastern part with Austria (considerably shorter section of the border; Fig. 2). In terms of the landscape, two parts of the Šumava National Park can be distinguished. In the north-western part there are large plateaus with emergent peaks and deep (but usually wide) valleys formed by fluvial erosion (in their upper parts most peatbogs are located). The south-eastern part in more varied in terms of the relief, including the Trojmezná mountain massif. It is where the highest peak of Šumava is located (Plechý, 1378 m). This part is surrounded from the north by the Vltava alluvial plain.

Adjacent to the Šumava National Park is the German Bayerischer Wald (Bavarian Forest) National Park, covering a much smaller area of 242.17 km² (Europarc-Germany 2011). Established already in 1970 (the oldest German national park), it covers a shorter section of the mountain range, corresponding to the landscape of the north-western part of the

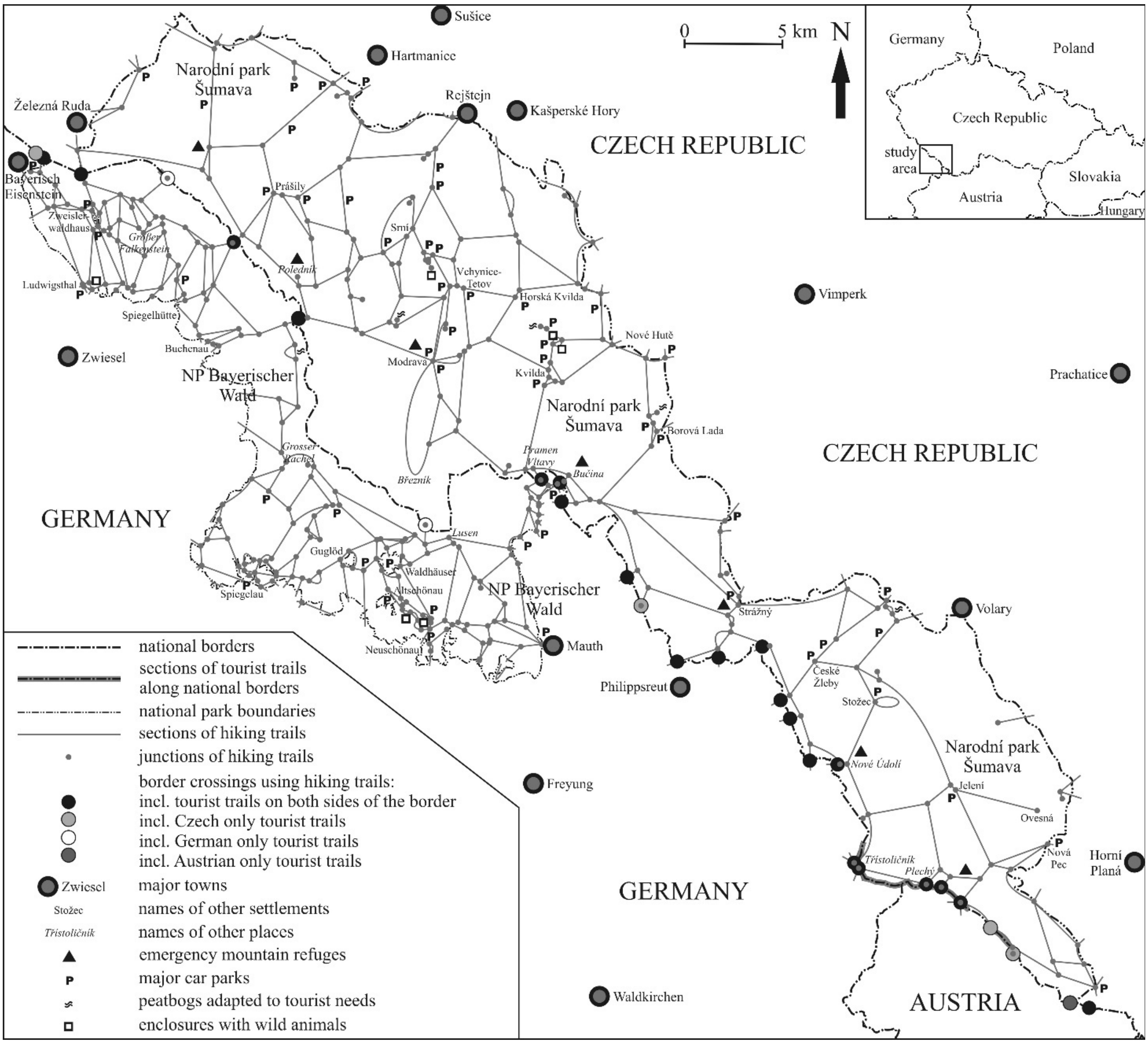

**Fig. 2** The network of hiking trails in the Šumava and Bayerischer Wald National Parks in 2019, including opportunities to cross the border and selected elements of tourism infrastructure.

Czech national park. The highest peak is Großer Rachel, 1453 m (the summit of the whole mountain range, Großer Arber, 1457 m, is located outside the national park). Both national parks described constitute one of the largest protected areas of this type in Europe. Within their borders the relics of primeval mountain forests, glacial lakes, as well as mountain and valley raised bogs can be found. Numerous species of animals rarely found nowadays in Central Europe, such as the lynx, black grouse, peregrine falcon, black stork, or the wolf, can be observed. On the other hand, the landscape provides various evidence of a quite intensive farming, which took place in the 19th and still at the turn of the 20th century (Breuer et al. 2010; Těšitel et al. 2003a). Today both national parks are a popular tourist destinations, however on both sides of the border it is mostly domestic (Dickie et al. 2014; Gorner and Čihař 2013; see also: Picek et al. 2007; Těšitel et al. 2003b). The Czech side alone is visited by approximately 2 million tourists every year, while the German – by 1.3 million.

Šumava from World War II to the political and economic transformation at the turn of the 1990s was largely inaccessible due to its location as a borderland (Breuer et al. 2010; Dokoupil and Kopp 2011). After 1945, Czechoslovakia very strictly guarded its border with the Federal Republics of Germany and Austria. Part of the mountain range was even transformed into a military training ground. The German-speaking population inhabiting these areas until World War II was displaced, and only to a small extent replaced by the Czech population (these were mostly military). The result was depopulation of the region, the disappearance of many settlements (some were even demolished) and secondary plant succession in former agricultural areas (Breuer et al. 2010; Těšitel et al. 2003a). It also stopped the development of tourism that had taken place here both before World War I, when this area was part of Austria-Hungary, and in the interwar period as part of Czechoslovakia. The Šumava National Park was established very shortly after the border policy changed and the theoretical opening of these areas to tourists at the turn of 1990s. As a result, mobility restrictions still remained (albeit to a lesser extent), not for political reasons but for nature conservation. The park authorities had a great influence on the development of the trail network which was adapted to the distribution of the most valuable areas. There were no such significant changes on the German side, except for gradual depopulation from the end of the 19th century as a result of better living conditions in urban areas. Nevertheless, the process of tourism development was not stopped and the German side of the mountain range was still quite popular (Mayer et al. 2010). A recent ecological phenomenon that influenced the landscape of the region and its tourist attractiveness was the large-scale impoverishment of coniferous forests as a result of air pollution and the weakening of trees by insect infestation (Krzystek et al. 2020; Šamonil and Vrška 2007; Vacek and Podrázský 2003; Zýval et al. 2016).

The Bayerischer Wald National Park consists of four protection zones: 1) nature zone (German: *Naturzone*) covers 72% (175.16 km², in 2027 it is supposed to be 75%), an area of strict protection without human influence on natural processes; 2) a development zone (German: *Entwicklungszone*), being a partial protection area; 3) a similar marginal zone (German: *Randbereich*), where active management is carried out to protect forests; 4) a recreation area (German: *Erholungszone*), which consists of areas with tourism infrastructure, including animal enclosures and information centers. In 2020, a new division into protection zones was introduced in the Šumava National Park with four categories in place of the previous three: 1) a natural zone (Czech: *zóna přírodní*; 27.7% of the park's territory, i.e. 189.5 km²), which is a strict protection area devoid of any human activity; 2) a 'close to nature' zone (Czech: *zóna přírodě blízká*; 24.5% of the park's territory, i.e. 168.2 km²), located in areas transformed by human activity which are to gradually return to their natural state; 3) a zone of 'concentrated care' for nature (Czech: *zóna soustředěné péče o přírodu*), occupying the largest part of the park and including anthropogenic areas, the maintenance of which requires active protection; 4) a cultural landscape zone (Czech: *zóna kulturní krajiny*), including areas used by people and built-up areas. This article, however, refers to the earlier division into three zones as it has influenced the development of tourism up to the present. This is all the more justified as the existing 'silent' zones (Czech: *klidová území*) correspond to the former extent of the core (first) zone with only a few extensions. It is the distribution of the 'silent' zones that has the greatest impact on opportunities for tourist penetration, because within them it is possible to move only along marked trails, while in other parts of the park access for tourists is virtually unlimited. As of 2020, the extent of the 'silent' zones has been increased from the former 9002 ha to 14,921 ha, i.e. from 13.2% to 21.9% of the park area.

## 4 Results

### 4.1 Public transport

The comparison of transport accessibility to both national parks must take into account their different areas and extension along the state borders. Taking into account public transport, in both cases it is possible to indicate parts where the dominant role, especially in terms of external accessibility, is played by a specific means of transport. The cross-border railway Klatovy – Železná Ruda – Bayerisch Eisenstein – Zwiesel – Gotteszell is important for the western parts of both national parks (Table 3). Interestingly, however, none of the passenger trains cross the border and Czech and German trains have integrated timetables at the border station Železná Ruda-Alžbětín/Bayerisch Eisenstein where the station building from 1877 is cut into two almost equal parts (Fig. 3A). Today it houses, among other services, a Czech-Bavarian information center to help planning visits to both national parks. Direct express trains to Prague indicate the importance of the Šumava tourist region on the Czech market (Vystoupil and Sauer 2012, 2017). On the German side, there is also the

**Table 3** The frequency of passenger trains on railway lines in the Šumava and Bayerischer Wald region (all year)

| Railway line | Number of runs on working days | Number of runs on weekends |
|---|---|---|
| Klatovy – Železná Ruda-Alžbětín | 10 | 9 |
| Volary – Lenora – Vimperk – Strakonice | 9 | 8 |
| České Budějovice – Horní Planá – Nová Pec – Černý Kříž | 7 | 7/8 |
| Číčenice – Prachatice – Volary – Černý Kříž | 8 | 7 |
| Černý Kříž – Nové Údolí | 11 | 11 |
| Bayerisch Eisenstein – Ludwigsthal – Zwiesel – Regen – Plattling | 16 | 15 |
| Zwiesel – Frauenau – Grafenau | 7 | 7 |

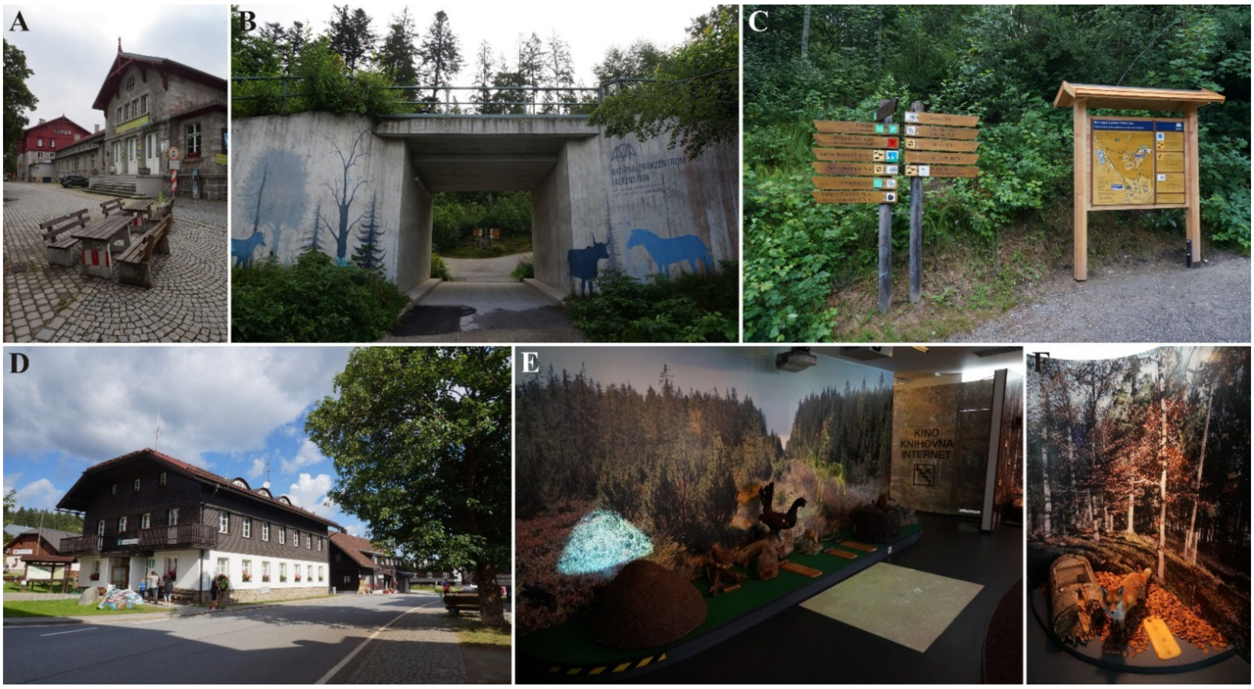

**Fig. 3** Selected visitor and information centers in Šumava and Bayerischer Wald National Parks: A. information point at the railway station Železná Ruda-Alžbětín/Bayerisch Eisenstein on the course of state border; B. entrance to the visitor center in Ludwigsthal from the car park, there is a railway line on the bridge and a halt is nearby; C. signposts and an information board within the visitor center complex in Ludwigsthal; D. a building housing an information point in the center of Kvilda; E. and F. shots of the exhibition in the Kvilda information point, to some extent the exhibition uses multimedia but generally it is rather simple (photos: author 2019).

local Zwiesel – Frauenau – Grafenau railway, the role of which is much smaller, but importantly has an almost completely tourist function (Table 3). The rest of the Bayerischer Wald National Park, both in terms of external and internal accessibility, is served by buses with departure points at Zwiesel, Frauenau, Grafenau and Freyung. The park is surrounded by a dense settlement network and all towns and villages can be reached by bus transport, although frequency depends on the location and season. The most extensive offer concerns villages in the surroundings of Zwiesel, north of Grafenau and the eastern edge of the national park (Table 4).

In Šumava National Park, railways are important in terms of external accessibility not only regarding its western edge, but also its eastern part. There are several lines with junctions at Černý Kříž, which is located within the national park, and Volary, which is less than one km from its boundary (Table 3). A railway line connects the national park with tourist centers on the Lipno reservoir, the largest artificial lake in the Czech Republic, widely used for recreation. However, contrary to the western part of the national park, in its eastern section there are no long-distance connections, so changes are necessary. In this case, the railway is also important for internal accessibility, as the lines on quite long sections run within the protected area boundaries or along its borders. At Nové Údolí station, trains reach an altitude of 800 m, making it possible to arrange hikes to the higher parts of Šumava on both sides of the border as the station is located very close to the border with Germany (it is a remnant of the cross-border railway line Černý Kříž – Waldkirchen, which on the German side was dismantled and transformed into a bicycle path). The locations of the railway stations are closely related to the network of tourist trails. The best example is the station in the almost depopulated settlement of Ovesná where trains stop precisely because of the high tourist numbers on a popular hiking and educational trail (Fig. 2).

The rest of the Czech national park (central and central-western parts) is served exclusively by buses (Table 5). The Šumava region is so popular for domestic tourism that many smaller villages located deep in the mountains are reached by direct long-distance bus lines from Prague, Plzeň and other cities of the Czech Republic. They are run by private companies so, as they are mainly driven by profit, the maintenance of these connections over several years demonstrates their profitability and the constant, and perhaps even growing, popularity of the presented area. Destination points are the main tourist centers of the region (Srní, Modrava, Kvilda, Borová Lada), but lesser known places (including Rejštejn and Vydra) are also served by these long-distance buses. Single journeys are maintained throughout the year, but in summer their number increases significantly (Table 5).

**Table 4** Bus connections from chosen settlements in the Bayerischer Wald National Park region

| Settlement | Working days* | | Weekends** | | Main directions |
|---|---|---|---|---|---|
| | AL | SE | AL | SE | |
| Altschönau | 15 | 3 | 15 | 2 | Finsterau, Grafenau, Mauth, Neuschönau, Passau, Riedlhütte, Spiegelau, St. Oswald |
| Bayerisch Eisenstein | 8 | 0 | 7 | 0 | Arber Bergbahn, Passau, Regen, Schönberg, Zwiesel |
| Buchenau | 4 | 2 | 3 | 1 | Lindberg, Spiegelhütte, Zwiesel |
| Finsterau | 6 | 0 | 6 | 0 | Altschönau, Mauth, Passau, Riedlhütte, Spiegelau, St. Oswald |
| Frauenau | 2 | 0 | 1 | 0 | Grafenau, Spiegelau, Zwiesel |
| Lindberg | 8 | 3 | 7 | 1 | Zwiesel, Buchenau |
| Ludwigsthal | 7 | 2 | 7 | 1 | Zwiesel, Zwieslerwaldhaus |
| Mauth | 7 | 0 | 7 | 0 | Altschönau, Finsterau, Passau, Riedlhütte, Spiegelau, St. Oswald |
| Neuschönau | 9 | 3 | 9 | 2 | Altschönau, Grafenau, Waldhäuser |
| Riedlhütte | 30 | 4 | 30 | 3 | Altschönau, Finsterau, Grafenau, Mauth, Neuschönau, Passau, Racheldiensthütte, Schönberg, Spiegelau, St. Oswald |
| Spiegelau | 32 | 2 | 26 | 0 | Altschönau, Finsterau, Grafenau, Mauth, Passau, Riedlhütte, Racheldiensthütte, Schönberg, St. Oswald, Zwiesel |
| Spiegelhütte | 4 | 2 | 3 | 1 | Zwiesel, Buchenau |
| St. Oswald | 9 | 2 | 9 | 2 | Altschönau, Finsterau, Grafenau, Mauth, Riedlhütte, Spiegelau, |
| Waldhäuser | 9 | 3 | 9 | 2 | Altschönau, Grafenau, Neuschönau |

**Note:** * means number of runs on working days in each direction; ** means number of runs on weekends in each direction. AL means all year. SE means seasonal.

**Table 5** Bus connections from chosen settlements within the Šumava National Park

| Settlement | Working days* | | Weekends** | | Number of cyclobuses (seasonal) | | Main directions |
|---|---|---|---|---|---|---|---|
| | AL | SE | AL | SE | Working days | Weekends | |
| Borová Lada | 5 | 4 | 1 | 4 | 4 | 4 | České Budějovice Horní Planá, Kvilda, Lenora, Netolice, Nové Hutě, Písek, Prachatice, Prague, Strážný, Tábor, Vimperk, Volary, Zdíkov |
| České Žleby | 4 | 0 | 0 | 0 | 0 | 0 | Stožec, Strážný, Volary |
| Dobrá Voda | 4 | 1 | 4 | 0 | 0 | 4 | Prášily, Sušice, Železná Ruda |
| Horská Kvilda | 1 | 6 | 1 | 5 | 4 | 6 | Kvilda, Modrava, Plzeň, Prague, Prášily, Sušice, Zdíkov |
| Jelení | 0 | 5 | 0 | 5 | 5 | 5 | Nová Pec |
| Jelenov | 7 | 0 | 4 | 3 | 7 | 7 | Kašperské Hory, Kvilda, Modrava, Sušice |
| Kvilda | 7 | 20 | 2 | 23 | 18 | 24 | Bučina, České Budějovice, Horská Kvilda, Netolice, Písek, Plzeň, Prague, Prášily, Srní, Sušice, Tábor, Vimperk, Volary, Zdíkov, Železná Ruda |
| Modrava | 7 | 6 | 5 | 10 | 12 | 15 | Domažlice, Klatovy, Kvilda, Modrava, Prague, Prášily, Sušice, Železná Ruda |
| Nová Hůrka | 3 | 7 | 4 | 7 | 6 | 11 | Domažlice, Klatovy, Kvilda, Modrava, Sušice, Železná Ruda |
| Prášily | 4 | 7 | 5 | 7 | 6 | 12 | Domažlice, Klatovy, Kvilda, Modrava, Prague, Sušice, Železná Ruda |
| Rejštejn | 10 | 1 | 5 | 6 | 9 | 9 | Kašperské Hory, Kvilda, Modrava, Plzeň, Prague, Sušice |
| Srní | 7 | 6 | 5 | 10 | 13 | 14 | Domažlice, Klatovy, Kvilda, Modrava, Prague, Sušice, Železná Ruda |
| Stožec | 3 | 0 | 0 | 0 | 0 | 0 | České Žleby, Strážný, Volary |
| Strážný | 6 | 2 | 1 | 1 | 2 | 1 | Borová Lada, Kvilda, Lenora, Vimperk, Zdíkov |
| Vchynice-Tetov | 7 | 7 | 5 | 10 | 13 | 15 | Domažlice, Klatovy, Kvilda, Modrava, Prague, Prášily, Sušice, Železná Ruda |

**Note:** * means number of runs on working days in each direction; ** means number of runs on weekends in each direction. AL means all year. SE means seasonal.

As for the local buses, their frequency is rather poor due to the small number of permanent residents and their routes are sometimes quite complicated to serve all settlements at the lowest possible cost. The result is that the journey can be long and hours of operation are adjusted to commuting to work or school, hence they are not attractive for tourists. In summer, however, they are complemented by typically tourist routes (Table 6).

Special tourist routes launched from the beginning of May until the end of September are characteristic for both national parks (cf. Kołodziejczyk 2014a). On the Czech side, as a supplement to the all-year routes being approximately meridional (from larger towns in the foreland to the mountains), they are rather latitudinal, along the course of the range connecting local tourist centers. All Czech tourist routes provide an opportunity for transporting bicycles (so-called ‘cyclobuses’), which reflects the great popularity of this form of active tourism and recreation in the Czech Republic and, at the same time, the potential of Šumava in this respect. In summer, the opportunity to transport bicycles also appears on selected all-year routes. Among the typical tourist routes, the one from Horská Kvilda through Kvilda to Bučina in the southern part is along a road with limited access, reaching the boundaries of the core zone, hence it is particularly popular. In the border hamlet of Bučina, it is connected with a German tourist route. Czech tourist buses run with a frequency up to every hour (although more often it is every 2–4 hours). There are many stops located outside settlements at the junctions of tourist trails or the starting points of educational paths. Czech tourist routes are also planned to connect car parks (Fig. 2), aimed at limiting the use of cars for moving around the park, but also making it easier for motorized tourists to plan their trips (in this way they do not have to be circular, no need to come back on foot to the starting point).

On the German side, tourist buses reach mainly unprotected enclaves located within the national park or connect settlements (including Buchwald, the equivalent of Bučina) at the eastern edge (Table 6). It is also possible to reach small, attractively located hamlets within the protected area, most often consisting of accommodation and catering facilities only where hiking trails start, as well as two mountain

**Table 6** The frequency of tourist bus lines in the Šumava and Bayerischer Wald national parks during the holiday season (July and August)

| Route | Number of runs in each direction | |
|---|---|---|
| | working days | weekends |
| Železná Ruda – Prášily – Srní – Modrava – Kvilda | 6 | 6 |
| Železná Ruda – Prášily – Hartmanice – Sušice | 1 | 4 |
| Horská Kvilda – Kvilda | 4 | 4 |
| Kvilda – Bučina | 7 | 7 |
| Nová Pec – Jelení Vrchy | 5 | 5 |
| Sušice – Kašperské Hory – Rejštejn – Srní – Modrava – Kvilda | 7 | 8 |
| Vimperk – Zdíkov – Nové Hutě – Kvilda – Borová Lada – Strážný | 2 | 1 |
| České Budějovice – Volary – Lenora – Borová Lada – Kvilda | 2 | 2 |
| Plzeň – Sušice – Rejštejn – Horská Kvilda – Kvilda | 1 | 1 |
| Spiegelau – Riedlhütte – Racheldiensthütte | 18 | 17 |
| Spiegelau – St. Oswald – Altschönau – Mauth – Finsterau – Teufelshäng | 7 | 7 |
| Grafenau – Neuschönau – Altschönau – Waldhäuser – Lusen, Waldhausreibe | 11 | 9 |
| Zwiesel – Lindberg – Buchenau – Lindberg – Zwiesel | 5 | 5 |
| Zwiesel – Lindberg – Ludwigsthal – Zwieslerwaldhaus – Ludwigsthal – Zwiesel | 8 | 8 |

car parks from where one can start a hike to Groβer Rachel (the first is located within the core zone, the second on its edge). There are minibuses running there, sometimes even using dirt roads. The frequency of the tourist buses on the German side is varied, sometimes these are just single journeys, but on the more popular routes there are several a day.

Comparing the transport accessibility of both parks, it can be concluded that it is better on the German side, but this is mainly due to the denser settlement network, higher number of permanent residents and the long and uninterrupted development of tourism. It seems, however, that the accessibility of the protected area is actually too good, because buses (and also cars) can reach many high-altitude locations (including within the core zone), thanks to which a large number of tourists can easily enter valuable natural areas. On the Czech side, public transport is less frequent and adapted to the road and settlement network. It is worth emphasizing that the offer significantly increases in the summer when the region is visited by numerous tourists. Czech buses also allow the interior of the mountains to be reached but to a lesser extent than in the German national park. The Šumava National Park authorities are also trying to limit car traffic by arranging car parks located outside settlements joined by bus routes that take tourists to a number of attractions and connect with the network of tourist trails (Fig. 2). Unfortunately, the offer is not as yet very popular and these car parks usually fill only partially, while at the same time there are problems with parking in the most popular places.

### 4.2 Hiking trail network and selected elements of tourism infrastructure

The hiking trail networks of the Šumava and Bayerischer Wald National Parks are unevenly developed, in the former much worse than in the latter, and the disproportion is noticeable not only between the two countries, but also locally within each of the protected areas (Fig. 2). These differences result from historical conditions and the principles of nature protection and tourism organization in each park. Compared to other protected mountain areas in Central Europe, the Bayerischer Wald has a well-developed network of trails, typical of the most popular regions with long traditions of mountain tourism (e.g. both national parks, Czech and Polish, in the Giant Mountains/Krkonoše/Karkonosze – Kołodziejczyk 2019; Styperek 2001), while Šumava can be compared with the Izerské or Orlické Mountains (Kołodziejczyk 2018), which are covered by less restrictive forms of nature protection (protected landscape areas) and are less popular among tourists. When making such comparisons, it is necessary to take into account the different surface of the protected areas. The Šumava National Park has exactly three-times fewer hiking trail junctions per km$^2$ (graph vertices) than the Bayerischer Wald National Park and a more than three-times fewer sections per km$^2$ (edges; Table 7). The exceptionally low density of junctions in Šumava (only 0.26 vertices/km$^2$) is noteworthy. In an area about three times larger than the neighboring German park there is a similar number of junctions (the difference is only 5.8%) and a 15% lower number of sections. Compared

**Table 7** Indices characterizing the hiking trail networks of national parks in the Czech-German borderlands in 2019

| Index | Values in individual national parks | | | |
|---|---|---|---|---|
| | Šumava | Bayerischer Wald | Bohemian ‘Switzerland’ | Saxon ‘Switzerland’ |
| Number of junctions (vertices) | 178 | 189 | 71 | 352 |
| Number of junctions per km² | 0.26 | 0.78 | 0.90 | 3.76 |
| Number of sections between junctions (edges) | 271 | 319 | 97 | 562 |
| Number of sections between junctions per km² | 0.40 | 1.32 | 1.22 | 6.01 |
| Kansky $\beta$ index | 1.52 | 1.69 | 1.37 | 1.60 |
| Kansky $\gamma$ index | 0.51 | 0.57 | 0.47 | 0.53 |
| Cyclomatic $\mu$ number | 97 | 133 | 29 | 213 |
| $\alpha$ index | 0.28 | 0.36 | 0.21 | 0.30 |

with the second pair of national parks in the Czech-German borderland, that is with Bohemian and Saxon ‘Switzerlands’, the networks of hiking trails are less developed (Table 7). In the case of both pairs, German parks are characterized by a higher density of trails. It is worth noting, however, that for the Bayerischer Wald National Park the density of junctions per km² (0.78) is lower than in the Bohemian ‘Switzerland’ National Park (0.90), which demonstrates that the network of tourist trails in the southern pair of parks is clearly less developed. By only taking into account the number of sections per km², the Bayerischer Wald is ahead, but only slightly, of Bohemian ‘Switzerland’ (1.32 vs. 1.22), but clearly behind Saxon ‘Switzerland’ (a difference of over 4.5 times).

All the graph indices (Table 7) show that the network is less coherent on the Czech side. While the degree of development refers to the length of the trail network, the coherence refers to the relationship between the number of junctions and sections of trails between them (that is vertices and edges). Differences between the two protected areas analyzed are clearly shown by the Kansky $\beta$ index and the $\alpha$ index, slightly smaller differences are revealed by the Kansky $\gamma$ index, which gives information about the proportion between actually existing and the potential number of sections resulting from the number of present vertices (in both parks it is just over half of the potential number). Therefore, on the German side, there are better opportunities for organizing mountain hikes, including circular routes, because the trail system includes many loops and there are more connections between junctions (Fig. 2). However, it should be emphasized that it is the less developed and less coherent Czech network that seems to best suit the tasks of a national park where the presence of people should be limited in general and made possible in selected areas only. The Šumava and Bayerischer Wald National Parks show a better network coherence than the pair of national parks in the north. In terms of each of the indices (Kansky $\beta$ and $\gamma$ index, and $\alpha$ index), the Šumava National Park has a more coherent network than the Bohemian ‘Switzerland’ National Park, similarly the Bayerischer Wald National Park has higher index values than Saxon ‘Switzerland’. At the same time, in terms of each index, Šumava (a Czech park with a more coherent network) is characterized by lower values than Saxon ‘Switzerland’ (a German park with a less coherent network). Of the four national parks in the Czech-German borderlands, Bohemian ‘Switzerland’ has the lowest cohesion, and the Bayerischer Wald, the highest. Taking into account the network classification developed by Taaffe and Gauthier (1973) and modified by Styperek (2001) (Table 1), the networks of hiking trails in both Šumava and Bayerischer Wald National Parks should be considered as a lattice system, although in the first case the value of the Kansky $\gamma$ index is very close to the lower limit of the range assumed for this type.

Despite the creation of new trails, the network in the Šumava National Park is still undeveloped and importantly it provides access to the core zone (the most valuable natural areas which are at the same time most susceptible to degradation; see: Křenová and Hruška 2012) to a very limited extent (Fig. 2). Thus, it can be said that in the Šumava National Park, the ecological function of hiking trails prevails over the tourist function and this cannot be said about its German counterpart. Formal marked routes only enter the core zone for short sections, usually being side-tracks (dead ends) from the main trail often taking the form of educational paths such as those leading to the peatbogs of Jezerní slať near Kvilda, Třijezerní slať near Modrava (Figs. 4D and 5B) and Chalupská slať near Borová Lada. The only longer sections within the core zone lead along selected valley floors (e.g. the upper part of the Teplá Vltava valley with the Vltava spring, parts of the Vydra valley or the Modravský stream valley) and the area of the Plechý peak and nearby Lake Plešné, which best represents the postglacial landscape. These are popular with visitors but are also used for educational purposes. The range of cycling routes entering the

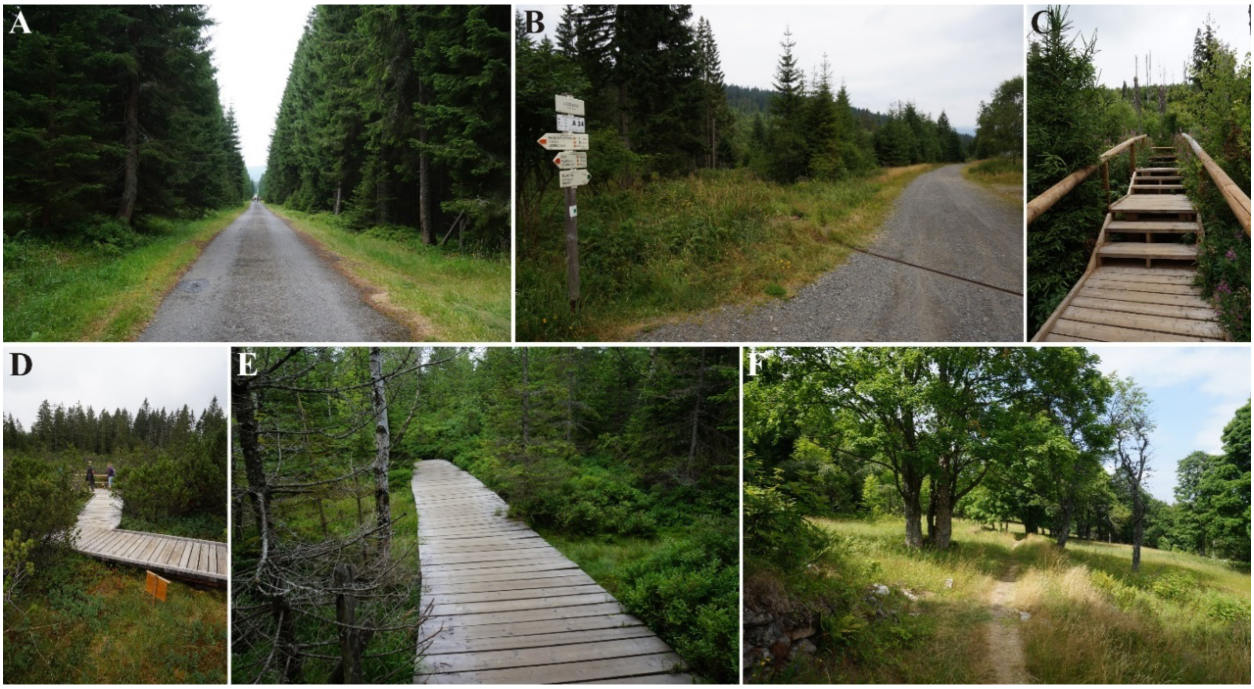

**Fig. 4** Types of roads and paths used by hiking trails in Šumava National Park: A. monotonous asphalt forest road near Modrava; B. forest track with a gravel surface in the Jezerní valley near Prášily, below Lake Prášilské; C. wooden boardwalks and steps leading to the Vltava spring; D. wooden boardwalk at the Tříjezerní slať peatbog near Modrava; E. section of the main red-marked trail on a boardwalk crossing a small wetland below Lake Prášilské; F. natural path being a former track through the depopulated village of Zelená Hora (photos: author 2019).

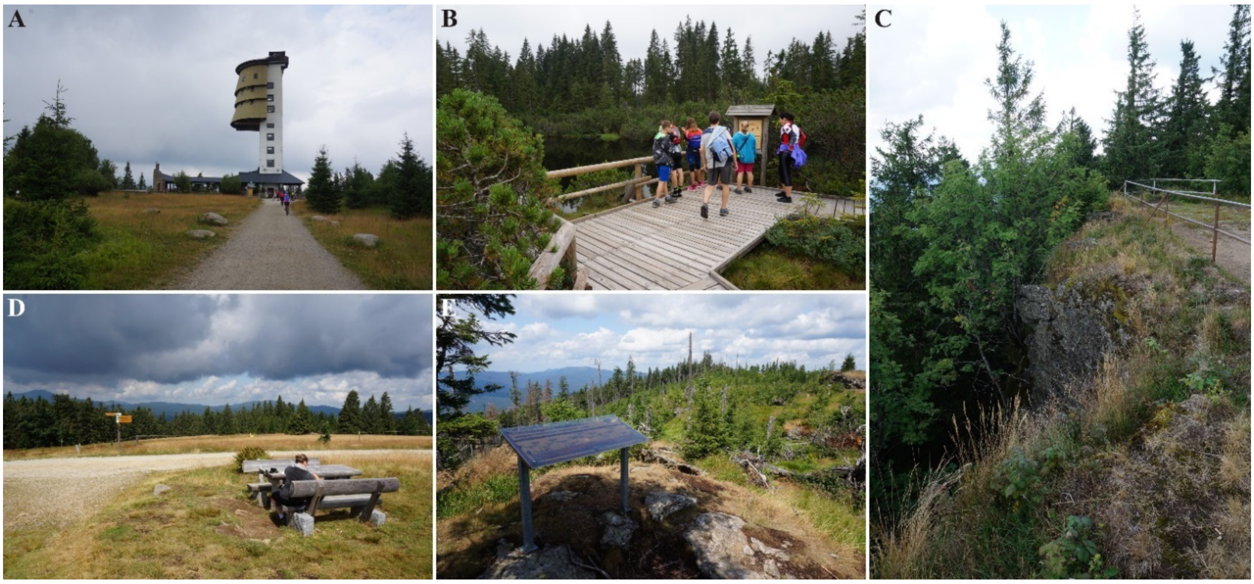

**Fig. 5** Places dedicated to admiring views in Šumava and Bayerischer Wald National Parks: A. view-tower on Poledník, 1315 m, originally a radar station from the communist period, rebuilt and opened to the public in 1998; B. observation platform at the Tříjezerní slať peatbog near Modrava; C. viewpoint, equipped with a railing, on top of Großer Falkenstein, 1315 m; D. tourism infrastructure at one of the viewpoints on the access trails to Großer Falkenstein; E. description panel on Rukowitzberg, 1269 m (photos: author 2019).

core zone is even smaller. It is worth noting that trails running through the core zone usually lead along previously existing paved roads (Fig. 4A, B) which makes the influence of tourists even more limited (provided that they do not leave the trail illegally). It should also be noted that certain sections of trails are closed due to the protection of wintering or nesting birds in the period from mid-November or mid-March to mid-July (depending on the section), which overlaps quite significantly with the holiday period. As a consequence, nature protection has priority over the development of tourist functions in the region. No long-distance routes of European importance pass through the park, and international (cross-border) trails are only of local character.

The dominance of hardened surfaces (asphalt, concrete, gravel) is one of the key disadvantages of the Šumava National Park's hiking trail network from the perspective of hikers (Fig. 4A, B). The entire mountain range was cut by a fairly dense network of forest roads, which were used for forestry and border control during the communist period. After the transformation and the creation of the national park, these roads were used to mark tourist trails because they did not require major expenditure, while the old routes from before World War II have often simply disappeared. The park authorities approved such practice, as it limited tourist pressure. Unfortunately, such surfaces have a negative effect on feet and make hiking monotonous (Frankowski 1966; Gajewski 2007). Sections with a natural surface are short, making up a small percentage of the entire length of the hiking trail network (Fig. 4F), occurring more often in the case of side-tracks leading to certain attractions (e.g. Oblík viewpoint, 1225 m; access to the glacial Lake Prášilské). The share of natural surfaces in the eastern part of the national park is slightly higher, with some sections of several kilometers (e.g. the yellow hiking trail from Jelení to the Ovesná railway station – 5.5 km; Fig. 2). Due to the nature of the surface of hiking trails, bicycle routes quite often overlap with them, which additionally reduces the convenience and safety of hikers. It must be said that Šumava is particularly popular among cyclists, which is reflected in a fairly developed network of marked bicycle routes and the above-mentioned offer of cyclobuses. Unfortunately, bicycle tourists often ride off the intended (official) routes. Regarding the surface of the hiking trails, it is also worth mentioning the wooden boardwalks over peatbogs open to the public and sometimes connected to observation platforms (Jezerní slať and Soumarské rašeliniště; Fig. 4D, E).

Educational paths are a specific type of hiking trail and there are 15 of them in the Šumava National Park, most of which follow regular hiking trails. Among them, the Bear's Trail deserves attention (Czech: *Medvědí stezka*, from Černý Kříž via Jelení to Ovesná; Fig. 2) which is the oldest trail of this type in the national park (and one of the oldest in the country) and at a length of 15 km presents various rock formations in its eastern part. The longest is the educational path devoted to the history of the Schwarzenberg canal [1] (Czech: *Naučná stezka Schwarzenberský plavební kanál*) which stretches for 30 km and partially extends beyond the park. Independent educational paths (not following regular hiking trails) were created for instance at peatbogs, although they are usually quite short (e.g. the educational trails Tříjezerní slať – 120 m, Chalupská slať – 440 m, Soumarské rašeliniště – 1.4 km). The Jezerní slať trail near Kvilda (330 m long) is an interesting case, as it is in no way connected with the rest of the hiking trail network, and its starting point can be reached by bike (cycling trail), car (car park) or local bus. Regular hiking routes do not coincide with paths leading to enclosures for wild animals (lynx and deer near Kvilda and wolves near Srní). These enclosures, organized by the national park authorities and available free, are to familiarize tourists with the natural living conditions of selected animal species, and at the same time reduce tourist footfall in the most valuable parts of the park by offering alternative attractions in easily accessible places with higher tourist resistance. Tourists go through the wolf enclosure entirely on wooden boardwalks while lynxes can be admired from a platform, but only the deer enclosure is crossed by a path.

Among the elements of infrastructure related to tourist trails, attention should be paid to seven emergency mountain refuges where a night can be officially spent (Fig. 2). As the Šumava mountain range and the national park are extensive with a sparse settlement and road network, it may happen that a

[1] Schwarzenberg canal (Czech: *Schwarzenberský plavební kanál*) is unique connecting one of the tributaries of the Studená Vltava with the Austrian tributary of the Danube, thus connecting the North and Black Sea basins. The canal was built between 1789 and 1822 and was one of the most successful technical structures on the Schwarzenberg estates. It served its intended purpose, floating wood, for almost 170 years (1793–1962).

tourist will not be able to reach their destination: an accommodation facility or a public transport stop. So there is an opportunity to spend a night within the national park borders in specially designated places, and these are fenced, equipped with tables, benches, litter bins, and usually with a toilet. This is an exception to the rule that tourists cannot stay in the national park and have to spend the night in an accommodation facility. The refuges are located far from villages and other tourism infrastructure, but at the same time close to attractive places and trail junctions (Hůrka u Prášil, Poledník, Modrava, Bučina, Strážný, Nové Údolí, Pod Plešným jezerem). In practice, however, they are used not only in emergency situations, but also by tourists who want to spend a night 'in the wild' which is tolerated by the park authorities. It is even noticeable that they are placed at distances that can be covered in one day enabling multi-day backpacking trips along the entire range.

The network of hiking trails in the Bayerischer Wald National Park is much more developed than on the Czech side (Fig. 2) and enters the core zone to a greater extent. In the latter respect, this is due not only to the considerable length of marked trails in the park and their course, but also to the greater proportion of the strict protection zone in the total park area and its spatial distribution. The *Naturzone* stretches along the entire park from the northwest to the southeast, along a fairly wide strip adjacent to the state border. Therefore, this zone must be crossed by all trails that connect the German and Czech networks, as well as by routes leading to the main natural attractions located in the higher parts of the mountains including the main peaks (Groβer Rachel; Groβer Falkenstein, 1315 m; Lusen, 1373 m). There are, however, fewer trails in some parts of the *Naturzone*, especially near the state border which is partly a relic of its former closure. The largest such wildlife refuges are located in the central part of the national park and at its eastern end.

The Bayerischer Wald National Park hiking trail network can be divided into two parts: north-western and south-eastern. The first covers a smaller area but is denser and they are separated by a low-density area above the reservoir near the town of Frauenau. Both parts are connected here by only one marked hiking trail (Fig. 2). The highest density is characteristic for the Groβer Falkenstein slopes and the areas near the boundaries of the national park, in the neighborhood of the surrounding villages and those situated in unprotected enclaves within the park (Fig. 2). There are numerous walking routes in their vicinity (e.g. the surroundings of the villages of Altschönau, Spiegelau and Zwieslerwaldhaus), some of a circular character. In their case, junctions with other trails are located every few hundred meters, and signposts showing the way to individual attractions or parts of settlements can be placed even more often. The density of trails in the national park is slightly lower than in the mountainous areas further to the northwest and southeast. This is an advantage in view of the principles of nature protection, however the density of tourist trails within the national park should still be considered definitely too high.

Long-distance routes run through the German park (the European Long-Distance Trail E6 and the partly overlapping *Goldsteig* route), which may result in increased numbers of tourists. Besides, the layout of the hiking trail network corresponds to proposed one-day or half-a-day circular trips leading from a village to individual peaks or to other tourist attractions, usually with a stop in a mountain hut. Hiking trails are mostly natural but there are sections leading along forest roads, usually dirt or gravel, rarely asphalt. From the perspective of a hiker, this fact – together with the density – is the main advantage of the marked hiking trail network in the Bayerischer Wald National Park. In addition, there are short sections along boardwalks (over peatbogs, e.g. Schluchtergasse path above the peatbog surrounding Lake Latschensee near Frauenau) and using steps carved into the rock (e.g. access to the viewpoint on Groβer Falkenstein). However, these are exceptions to the vast majority of trails leading along ordinary forest roads and paths. More than a dozen viewpoints equipped with barriers have been organized, mainly in the massifs of the highest peaks (Groβer Rachel, Groβer Falkenstein; Fig. 5C, D, E).

In the south-western part of the national park, in the vicinity of Ludwigsthal, a large visitor center, Haus zur Wildnis (Figs. 2 and 3B, C), was created with an exhibition (partly using multimedia), a view-tower, animal enclosures with observation points, an artificial cave, a restaurant and a car park. Nearby, there is a railway station and a bus stop with routes to various parts of the park and to other attractions in its vicinity. The main disadvantage of the complex is its poor connection with the network of tourist trails, as only two hiking trails (and no bicycle route) lead from there into the protected area. The second such center is located in the south-eastern part of the park above the village of Neuschönau (Fig. 2) where the program is slightly less extensive (tourist information center, futuristic view-tower, animal enclosures, restaurant, café, car park, bus stop). However, in this case there are more numerous hiking trails, including one of a circular character (leading through all 15 animal enclosures which are one of the main attractions of the center). Both places are to be gates into the national park, but some people may end their sightseeing there because in each of them a few hours or even a whole day can be spent carrying out the suggested hiking trips around the adjoining area. There is a lack of such visitor centers on the Czech side (Fig. 3D, E, F). Among other elements of infrastructure in the German national park there are three mountain huts, but these are not found in the Czech park. Together with the accommodation and catering facilities in the villages located within and close to the national park, this makes the Bayerischer Wald National Park an area very well equipped with tourism infrastructure, much better than on the Czech side. Weatherproof sheds and rest zones are also numerous here and distributed quite evenly throughout the area. The network of hiking trails is connected to the car parks (Fig. 2) which are located both near settlements and in the interior of the mountains (e.g. the two car parks on the southern slopes of Groβer Rachel already mentioned or the Waldhausreibe car park below the Lusen peak).

## 4.3 Tourist hubs and the main tourist flows

In the region of the Šumava and Bayerischer Wald National Parks, there are no large relief landforms such as great river valleys, that would predetermine tourist movements (the upper Vltava valley in the east is an exception). Therefore, the directions of tourist movements relate primarily to the transport network, railway lines and roads. The main directions are roughly concentric (Fig. 6), connecting larger towns (on the Czech side: Plzeň, Klatovy and Sušice in the north, Tábor, České Budějovice, Prachatice and Vimperk in the northeast, and Český Krumlov in the east; on the German side: Passau, Freyung and Grafenau in the south, and Plattling, Deggendorf, Regen and Zwiesel in the southwest) with the main tourist hubs and centers serving both national parks. The only longer and more significant cross-border route is the one from Regen via Zwiesel, Bayerisch Eisenstein, Železná Ruda to Klatovy, which corresponds to the railway line, but traffic across the border is much less than domestic. The other sites of cross-border traffic are Bučina/Buchenau, the area of Strážný and Philippsreut and Nové Údolí/Haidmühle; the latter two sites no longer included within the German national park. Apart from the first case, trips across the border are few.

Among the settlements in the vicinity of Šumava National Park, the following should be considered as primary tourist hubs influencing tourist movement the most (Fig. 6; Table 8): 1) Železná Ruda, from which tourists head towards the surrounding ski resorts and the natural assets of the western part of Šumava, only partially covered by the national park (tourist movement is more to the west than to the east of the town); 2) Sušice from where buses depart to the western and central parts of the park, including the most important local tourist centers (Srní, Modrava, Kvilda); 3) Vimperk which is a transfer point for tourists travelling from inside the Czech Republic, including Prague, to the central part of the national park (mainly to Borová Lada and Kvilda); and 4) Prachatice, located on the route from České Budějovice to the central and eastern parts of the park. As mentioned above, it should also be remembered that more distant towns play a significant role in the distribution of tourists. The role of Český Krumlov should be emphasized here which, as one of the most popular tourist destinations in the Czech Republic (Vystoupil and Šauer 2012, 2017), also generates tourism towards Šumava. Numbers of tourists arriving from the eastern side are additionally increased by those staying by Lipno reservoir where several campsites, hotels and numerous guesthouses operate. Secondary tourist hubs are located near the national park borders constituting transfer points between local buses (in the east, there is also a connection with the railway in Volary, Nová Pec and Horní Planá; Table 8). From these places, tourists go to specific attractions within the boundaries of the national park.

Most of the local tourist centers in Šumava National Park can now be considered as fully formed (existing; Table 8), although this is the result of a number of private and public investments in recent years. It is a response to a specific fashion for Šumava that developed among Czechs at the beginning of the

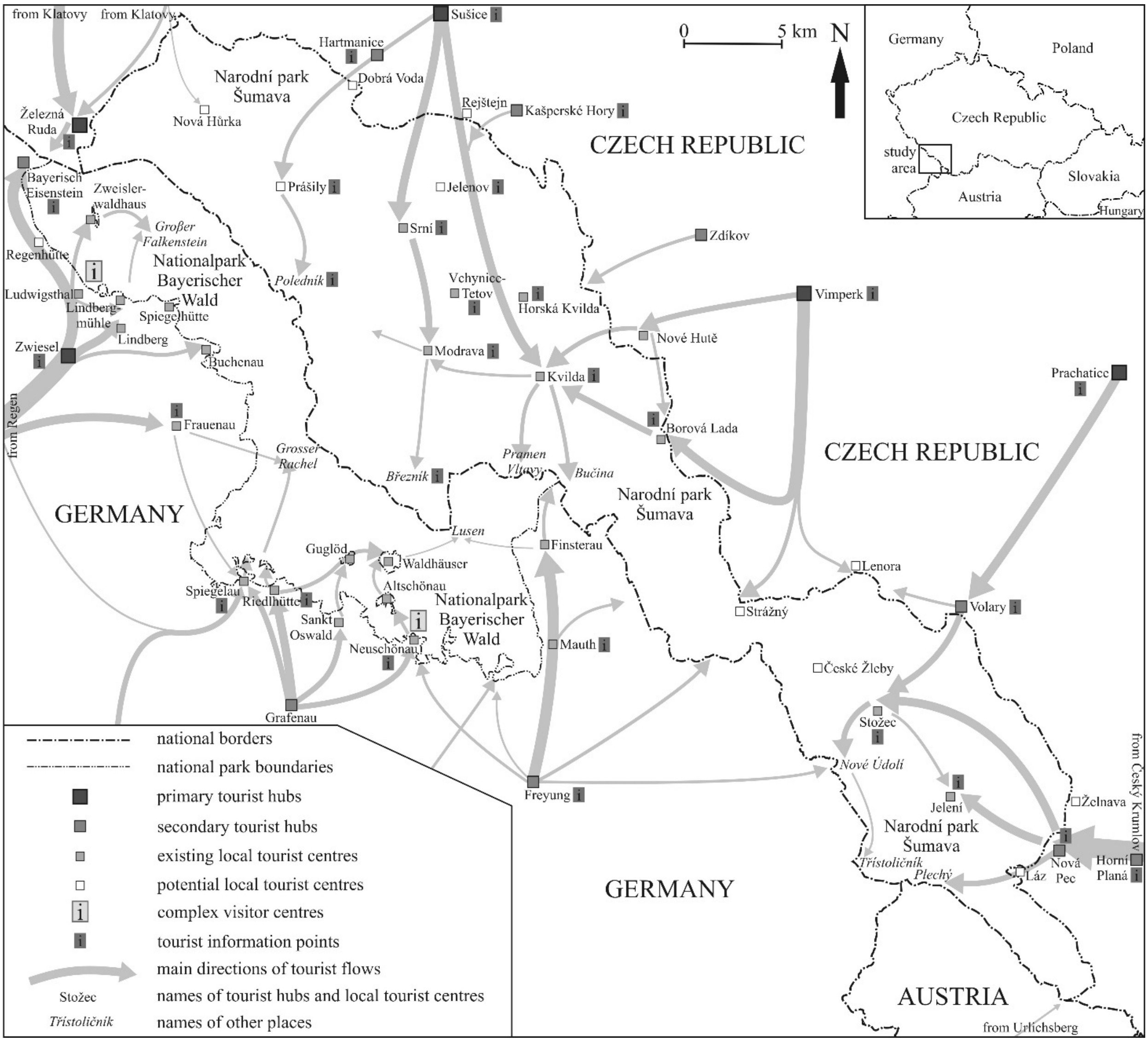

**Fig. 6** Chosen aspects of tourism management in the Šumava and Bayerischer Wald National Parks.

**Table 8** Tourist centers serving the Šumava and Bayerischer Wald National Parks

| Hierarchical level | Šumava National Park | Bayerischer Wald National Park |
|---|---|---|
| Primary tourist hubs | Prachatice, Sušice, Vimperk, Železná Ruda | Zwiesel |
| Secondary tourist hubs | Hartmanice, Horní Plana, Kašperské Hory, Nová Pec, Volary, Zdíkov | Bayerisch Eisenstein, Freyung, Grafenau |
| Existing local tourist centers | Borová Lada, Horská Kvilda, Jelení, Kvilda, Modrava, Nové Hutě, Srní, Stožec, Vchynice-Tetov | Altschönau, Buchenau, Finsterau, Frauenau, Guglöd, Lindberg, Lindbergmühle, Ludwigsthal, Mauth, Neuschönau, Riedlhütte, Sankt Oswald, Spiegelau, Spiegelhütte, Waldhäuser, Zwieslerwaldhaus |
| Potential local tourist centers | České Žleby, Dobrá Voda, Jelenov, Láz, Lenora, Nová Hůrka, Prášily, Rejštejn, Strážný, Želnava | Regenhütte |

21st century[2] (Vystoupil and Šauer 2012) that in a

[2] The popularity of Šumava increased even more during the COVID-19 pandemic which resulted from restrictions on trips abroad and on the functioning of tourist infrastructure (accommodation and catering). The mountains experienced a peculiar raid of caravans that did not fit into campsites and parking lots, as a result they stood along the roads, sometimes even making it difficult to pass.

relatively short time in some villages accommodation and catering facilities were developed on a fairly large scale. Nowadays the accompanying facilities (tourist information points, educational panels, equipment rentals, etc.) are trying to follow this process. Existing tourist centers are located in the middle of the park (Srní, Vchynice-Tetov, Modrava, Kvilda, Horská Kvilda, Borová Lada; moreover, Nové Hutě, partly connected with down-hill skiing in the immediate vicinity of the park), and on the eastern edge, under the influence of Český Krumlov and the Lipno reservoir (Jelení, Stožec). Between these two areas as well as on the western edge of the park there are potential centers (Fig. 6; Table 8), although the infrastructure developing there sometimes results in conflicts with the local community. For example, in Prášily, the local population protests against the large number of parked cars, especially as there is a large car park at Poledník Slunečná one km away. Both places are linked by a regular bus route. It is from this car park and the local bus stop that tourists are supposed to set off to the summit of Poledník, 1315 m, with a view-tower and a national park information point (Fig. 5A). This car park is a very good example of preparing a starting point for tourist trips (cf. Kołodziejczyk 2015a, 2015b).

The directions of tourist movements within the Šumava National Park correspond to its most popular sections (Fig. 6). The route up the Vydra river, from Rejštejn via Srní to Modrava, is important, with an extension (only on foot or by bike) deep into the mountains to the Březník settlement where tourists can find a small exhibition and a restaurant (these are the only buildings there, the remnant of a former village). The second important route leads from Rejštejn through Horská Kvilda to Kvilda, however, this local tourist center is often reached from the east. A popular destination for excursions from Kvilda is the already mentioned Bučina and the Vltava spring (more precisely, it is Teplá Vltava), which is of great importance to Czechs for patriotic reasons (Fig. 4C). In the eastern part of the park, many tourists travel up the Vltava valley from the Lipno reservoir to Nové Údolí, and to the mid-forest settlement of Jelení. The main directions of tourist movements within the Šumava National Park are shaped primarily by the layout of public roads and the offer of public transport, but in the interior of the park, especially in its central part, the importance of the distribution of tourist attractions and infrastructure, including the network of trails, is increasing. The location of the national park information points (including Prášily, Modrava and Kvilda; Fig. 3D, E, F) has a limited influence on these directions, especially as some of them are already deep in the mountains (Poledník, Březník, Jelení). They are most often visited by tourists spending their holidays in the surroundings hence they play a role in shaping the directions of short trips from a given location.

The German national park is smaller and the number of main tourist routes is lower (Fig. 6). The western part is most often reached from the southwest (from the city of Regen) by rail or individual transport. From Zwiesel, the primary tourist hub in this area (Table 8), visitors go to individual local tourist centers at the foot of the mountains, with Lindberg and the adjoining hamlets playing the main role (due to the number of beds in accommodation facilities), moreover, Ludwigsthal (due to the large national park visitor center, where starting a visit to the national park is recommended) and Bayerisch Eisenstein, which is a secondary tourist hub, although servicing areas mainly further west and to a lesser extent both German and Czech national parks. From Regen, tourists also head to Frauenau and its individual administrative districts, from where some go to the eastern section of the park. For this section, it is problematic to indicate the main hubs of tourist movement (except for Zwiesel, which is peripheral). This is due to the complicated system of roads leading from the interior of Bavaria, the lack of main railway lines and a larger urban center (transport junction) in the eastern foothills of the Bavarian Forest. Such a function could only be attributed to Passau and Deggendorf, located several dozen kilometers south. There are, however, two localities that play the role of secondary tourist hubs (according to the classification in Table 2), Freyung and Grafenau, which are reached by local, insignificant railway lines, hence the greater role of bus transport.

Due to the lack of main tourist hubs, the directions of tourist movements to the eastern part of the Bayerischer Wald National Park are approximately parallel to each other, corresponding to the roads leading to individual settlements. Among the attractions inside the protected area, the most popular are two peaks: Groβer Falkenstein, which is usually hiked from Lindberg, Ludwigsthal and Zwieslerwaldhaus; and Groβer Rachel, which is climbed from Frauenau, Spiegelau and Riedlhütte. Due to the long tradition of tourism in the Bavarian Forest, almost all local tourist centers (within the borders and in the immediate vicinity) can be considered developed (Table 8). They offer diverse accommodation, catering and accompanying facilities, and are efficiently connected by the network of tourist trails. Tourism functions are particularly dominant in smaller villages and hamlets that are unprotected enclaves within the national park.

## 5 Discussion

Compared to other protected areas in Central Europe, the Šumava and Bayerischer Wald National Parks as a whole are characterized by a moderate degree of development and a fairly good coherence of hiking trail networks, with the results when analyzed separately, being much higher for the German park. Their network coherence is lower than in both parks in the Giant Mountains (e.g. for the Czech Krkonoše National Park, the Kansky $\beta$ index is 1.84, the Kansky $\gamma$ index 0.61, and the $\alpha$ index 0.44; Kołodziejczyk 2019), but higher than in the Czech Izerské Mountains Landscape Park (Kansky $\beta$ index is 1.40, Kansky $\gamma$ index 0.47, and $\alpha$ index 0.21; Kołodziejczyk 2018). As mentioned above, taking into account the network classification presented in Table 1, the networks of hiking trails in both the Šumava and Bayerischer Wald National Parks belong to the lattice system. For comparison, the network in the Krkonoše National Park should also be classified as a lattice system, while in the Izerské Mountains Landscape Park it is the less coherent core-lattice system (Kołodziejczyk 2018, 2019). Compared to Polish national parks, the hiking trail networks of the Šumava and Bavarian Forest should be considered very coherent and fairly well-developed (cf. Styperek 2001). In terms of the density of the trail network, the two parks can be compared with the Kampinos, Karkonosze (Giant Mountains) and Stołowe (Table) Mountain National Parks, and the even more extensive network in the Tatra National Park. The above relations confirm the impact of the tourism history on infrastructure development, including the network of tourist trails. The Giant and Tatra Mountains are among those areas where organized tourism began to develop earliest in Central Europe (Lindrová 2018; Lokvenc 2006; Maziarz et al. 2017; Potocki 2004, 2010; Stastna 2006). They are characterized by very attractive postglacial landscapes (Boltižiar 2009, 2010; Drewnik et al. 2008; Pilous 2016) which are unique in the region causing considerable interest from tourists. The networks of marked hiking trails in these two ranges had already begun to develop in the 19$^{th}$ century and today are among the densest in the Polish mountains. Similar in character and the history of tourism development is the Bavarian Forest where an advanced network of hiking trails and walking routes around mountain tourist settlements has been created.

In each case (the Bavarian Forest, the Giant and Tatra Mountains), the network of tourist trails should be considered overdeveloped for national parks, preventing the implementation of their basic protective functions, e.g. due to the fragmentation of the environment and the lack of a sufficiently large refuge for animals away from human disturbance. In these parks, tourist routes sometimes intersect at less than 1 km, which means that the space in between, where human influence is theoretically limited, is less than 1 km$^2$ (but the impact still exists, e.g. in the form of noise). The opposite is the Šumava National Park, where the nodes of tourist trails are located a few, sometimes even tens kilometers away. Combined with large areas completely excluded from tourist traffic, this creates much better conditions for the functioning of animals and the preservation of biodiversity. The areas without human influence in the Czech park are several square kilometers in size which ensures good conditions for the implementation of the protective function.

On the other hand, Šumava can be compared with other mountain ranges located in the border zones of the so-called Soviet bloc countries in Eastern Europe which were under the influence of the Union of Soviet Socialist Republics before 1990. Despite the official friendship between these countries, opportunities to stay in border zones were very limited, often requiring special passes (not to mention crossing the border). The result was a lack of tourism infrastructure development (Mazurski 2012; Sroka 2013), not only accommodation and catering facilities, but also tourist trails. For example, in the Polish Sudeten, where the border with Czechoslovakia ran at that time, for several decades after World War II, no trails between the border and the first public road were created (Dudziak and Potocki 1995; Kołodziejczyk 2014b). Even greater restrictions were in force at the borders with capitalist countries, i.e. in the case of Czechoslovakia in the Ore Mountains (Czech: *Krušné hory*), Šumava and in southern Moravia (Dokoupil and Kopp 2011) where often a fairly wide border zone was inaccessible, in practice being a deserted area patrolled by the army. The road networks in these areas were subordinated to military needs. Along a large part of the border of Czechoslovakia with Austria and the Federal Republic of Germany, there was a patrol road (so-called *signálka*), along which there were a series of barbed wire fences and a strip of ploughed soil ('no-man's land'), across which information was electronically signaled if an attempt to cross this zone was made. The road is clearly visible in the landscape even today but currently, the vast majority of its course is used for tourist purposes.

Limitations in the development of tourism in the communist period allowed the tourism infrastructure in national parks created immediately after these border areas were made available to the public to be made appropriately. An analogous example to Šumava is the Podyjí National Park (Kołodziejczyk 2015a, 2015b), located on the Czech-Austrian border near Znojmo. This protected area of 63 km² was established in 1991 (TRASA 2007). It covers the spectacular, 42 km long gorge of the Dyje river (between Vranov nad Dyjí and Znojmo), with slopes up to 150 m high, hence the valley is surrounded by mountainous landscape with significant height differences, while areas further away are a relatively level plateau. The degree of development of the tourist trail network in the Podyjí National Park corresponds to the high protection level. The density of trails is low, for example, there is only one hiking and cycling trail running through the central part of the park (Fig. 7). This is confirmed by the very low values of the graph indices (Table 9), clearly lower than in the case of not only Bayerischer Wald, but also Šumava. The number of edges is only slightly higher than the number of vertices, especially concerning the cycling network in which there are only two loops and the $\beta$ index is only 1.04. Both networks should be considered as core-lattice systems, however for the cycling network the value of the $\alpha$ index (0.04) almost means a core system.

So far, the problem of tourism organization in the region of Šumava and Bayerischer Wald has been addressed mainly by Czech authors (e.g. Křenová and Hruška 2012; Křenová and Kiener 2012), especially referring to the biosphere reserve established in this area (Kušová and Těšitel 2016, 2017; Kušová et al. 2008; Těšitel and Kušová 2010). On the one hand, it is an expression of the considerable importance attached to tourism development in the Czech Republic (cf. Bousset et al. 2007), including protected areas, on the other, a consequence of a series of controversies regarding management in the national park, including leaving forests in the core zone without any human interference when they were attacked by the bark beetle (Zýval et al. 2016). In this case, a discussion on the park's zoning was provoked which concerned both protection and tourism issues (cf. Křenová and Hruška 2012). Formal changes in this respect, as indicated above, took place in 2020, but were the result not only of analyses of the Šumava environment, but also changes in Czech law. Similar to the present work, many studies have analyzed the relationships between the national parks on both sides of the border, e.g. through the perspective of implementing international projects or involvement from local communities (e.g. Kušová and Těšitel 2016; Stemberk et al. 2016). The work carried out focuses mainly on the problems of national parks or the opportunities provided by tourism for developing peripheral areas (e.g. Těšitel et al. 2003a). However, little attention has been paid to the fact that the Šumava National Park can be a source of many positive patterns for tourism development in protected areas, which is what this article is doing.

The graph approach presented here seems to be an appropriate method of analyzing the development of trail networks in protected areas. It allows the network to be simplified (omission of the winding nature of the actual route) which makes it easier to identify the potential for organizing trips by tourists and to analyze the directions of their movement (stimulating their dispersion). In addition, the indices used in graph theory allow a network's density and coherence to be determined. The more coherent the network, the more sections of trails between junctions, and, consequently, the greater the segmentation of geographical space, and the greater the pressure of tourism on the environment. The layout of the trail network determines the directions of tourist movements (e.g. along the main route constituting the core of the network) and, consequently, ways of facilitating marked trails with various types of infrastructure. The graph approach seems to implement the position of Stasiak (1997) who pointed out that the indicators used to describe the tourist function of protected areas

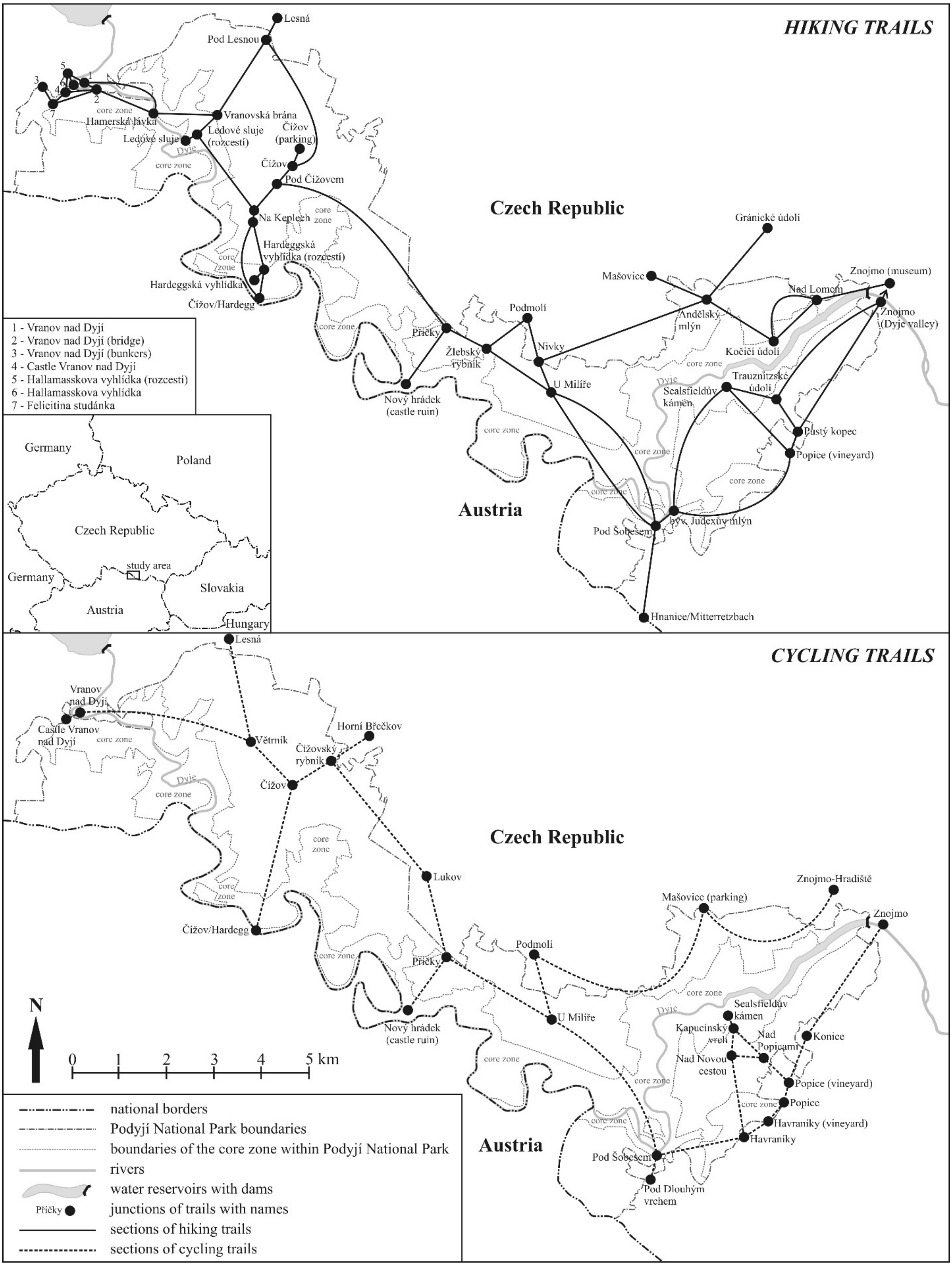

**Fig. 7** Networks of hiking and cycling trails in the Podyjí National Park as of 2014. Source: Kołodziejczyk 2015a, adapted.

**Table 9** The values of the cohesion indices of the tourist trail networks in Podyjí National Park as of 2014

| Index | Hiking trail network | Cycling trail network |
|---|---|---|
| Number of junctions (vertices) | 41 | 27 |
| Number of sections between junctions (edges) | 51 | 28 |
| Kansky $\beta$ index | 1.24 | 1.04 |
| Kansky $\gamma$ index | 0.44 | 0.37 |
| Cyclomatic $\mu$ number | 11 | 2 |
| $\alpha$ index | 0.14 | 0.04 |

Source: Kołodziejczyk (2015a).

(e.g. the number of tourists per km² in a park, or kms of trail) are purely theoretical, as they represent average values, while, in practice, the distribution of tourists varies greatly, even within a small area. Some places (and trails) are relatively rarely visited, while others are overcrowded and ecologically threatened. Therefore, this article has analyzed the spatial diversity of the tourist trail network as well as the intensity and direction of tourist movements. In this way it is possible to identify areas that are less or more penetrated. Stasiak (1997) also emphasized that in order to avoid a concentration of tourists in certain areas, it is necessary to skillfully manage the flow of tourists.

## 6 Conclusions

The ways in which tourism is organized in the Šumava and Bayerischer Wald National Parks differ significantly. The availability of geographical space for tourists is much greater in the second, and the tourism infrastructure is clearly more extensive there including the tourist trail network, mainly due to the longer and uninterrupted development of tourism. Considering the main functions of protected areas, it can be assessed that on the German side, the protective function is dominated by the tourist one, while on the Czech side, the protective function is still the most important. Similar relations occur in the case of tourist trail functions; in the German park, their ecological function is overshadowed by making space available for tourists. On the contrary, Šumava can be identified as a model in terms of adjusting the directions of tourist movement to the needs of environmental protection, therefore the hypothesis from the beginning of the article is confirmed. However, whether the growing popularity of this area will have a negative impact on the relationship between the protective and tourist functions should be considered, especially during the COVID-19 pandemic and increasing inbound tourism. The number of accommodation and catering facilities is increasing, alongside vehicular traffic, and tourists do not necessarily want to use solutions that would limit their negative impact (a network of car parks and tourist bus routes). Caravans, which are more and more popular in the present situation, also have a negative impact on the environment (e.g. sewage, garbage). It is also necessary to provide them with properly equipped parking spaces and sewage disposal sites. The network of tourist trails remains poorly developed, but pressure for creating new routes, especially for cycling, is clearly noticeable. This is also reflected in the increasing number of educational trails which, however, are created in areas more resistant to human pressure, and which at the same time can shape pro-ecological attitudes.

Decisions connected with the management of a network of tourist trails (both its development and limitation) should be in agreement with conservation rules valid in particular zones within protected areas (cf. Křenová and Hruška 2012). A good example of this is the Šumava National Park, which, thanks to previous access restrictions in the communist period could be perfectly developed for tourism in the 1990s (the park was established in 1991, very soon after opening of this area to wider public). This process was similar in another Czech national park – Podyjí. The total length of tourist trails within national parks should be minimalized as far as possible, although it is particularly important for their most valuable parts (usually referred to as the core zone). Certain attention should be paid to areas of strict protection that are elongated and narrow (e.g. river valleys or mountain ridges) as even a short section of trail may be a serious threat. Trail entering the core zone should be dead-end or U-shaped thanks to which these areas will have the lowest possible accessibility level (Fig. 8). Tourism infrastructure in such sections should be minimal (at most benches and water supply), while a more developed infrastructure (rest zones with benches and tables, weatherproof sheds, tourist hostels) is highly inappropriate. It should be created outside protected areas or, which is less desirable, within national parks, but outside strict protection zones. Monotonous sections should be avoided, e.g. along asphalt forest roads without any bends or views, as this may make tourists want to leave the trail. Such illegal and uncontrolled dispersion may cause more damage (cf. Kolasińska et al. 2015) than allowing tourists into a narrow zone of a tourist trail, even if it runs through the most valuable parts of a protected area. Whenever possible, hiking trails should have a natural surface (clay, gravel, stones, etc.) because tourists in national parks look for experiences that are different from the everyday.

In both national parks analyzed, even in the Bayerischer Wald, despite its disadvantages in terms of general tourist management, certain solutions (patterns) can be identified which, after appropriate adaptation, may bring positive effects in other protected areas. These include:

(1) Organizing discovery (visitor) centers within or near national parks, functioning as 'gates' to protected areas. They should provide tourists with comprehensive information on nature conservation rules, the main protection aims in a given park and most important tourist values of the geographical environment. In the same time they should have an interesting form, providing entertainment, and in this way attracting tourist to visit this facility instead of some vulnerable natural area prone to human-related damage. Therefore, the program of such facilities must be really varied, including not only an exhibition (multimedia elements desired), but also i.a. open-air attractions, animations for children or a view-tower. The location of discovery centers must be connected with the main flows of tourists to (or within) a park (as in Bayerischer Wald), or on the contrary, they could be a kind of a magnet attracting tourists to less popular places (but still resistant to anthropopressure).

(2) Providing other extra attractions in less valuable areas capable of absorbing more tourists. Places such as enclosures with wild animals, organized observation platforms, and viewpoints and towers should be located rather outside the national park. They can become specific buffers attracting tourists and in the same time lowering the pressure on the protected area, especially core zones.

(3) Opening tourists to such places that are attractive and at the same time easily accessible, e.g. located near public roads thus leaving similar spaces that are located deep in the mountains in the interior of the protected area closed. This idea is very well implemented in the Šumava National Park where four easily accessible peatbogs have been adapted to tourist needs (educational paths, wooden boardwalks, observation platforms), and much larger complexes, e.g. the valleys of the Studená Vltava, Modravský and Roklanský streams, remain inaccessible or can be viewed to a limited extent only from the boundaries of the core zone.

(4) Facilitating access to particularly popular places, where, for various reasons, there are no prospects of limiting the influx of tourists (e.g. the Vltava spring, summits with viewpoints and view-towers). As tourists will always go to such places, their negative impact should be limited as much as possible, both on the access way and at a specific attraction. A good example of this are the wooden boardwalks and steps prepared in the vicinity of the Vltava spring in the Czech national park or more extensive tourist

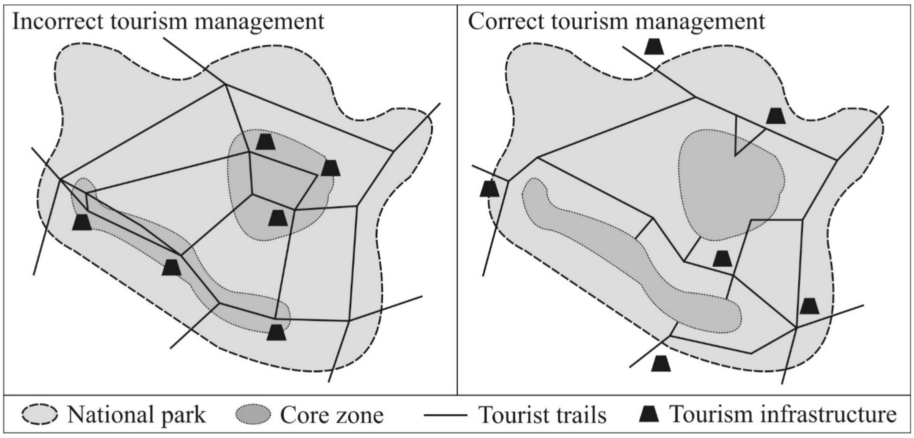

**Fig. 8** Models of tourist infrastructure development in national parks.

facilities on the access trails to Großer Falkenstein on the German side.

(5) The temporary exclusion of certain parts of national parks from tourists due to protection needs, e.g. during the bird breeding season as in the Šumava National Park.

(6) Regarding tourist infrastructure, in the protected areas, especially in core zones, only tourist trails (hiking ones most desirable), facilities providing information and educational boards should be developed. The idea is more fully implemented in the Šumava National Park which was created in an area that was largely unavailable for about half a century, than in the Bayerischer Wald National Park.

(7) Public transport should meet tourists' needs regarding time and space. In more popular areas it is necessary to launch additional seasonal connections on particularly important or attractive routes in order to encourage some tourists not to use their car during vacation. In planning the development of public transport the absorption (capacity) of the area must be taken into consideration so that too many tourists delivered to a certain place do not cause damage. Routes and bus stops should be connected with the network of tourist trails, the most typical starting points for hiking and cycling trips, and the location of car parks, so that the offer is attractive for motorized tourists as well and encourages them to limit, at least partly, their use of a car.

The presented examples of national parks show that history has a great influence on tourism management. As noted by Kołodziejczyk (2020d, p. 2876):

*Protected areas created in locations which have so far been unpopular with tourists, where infrastructure was developed only to a limited degree (or not at all), may be developed fully according to environmental protection rules. In all other cases the former infrastructure, directions of tourist movement and the network of popular attractions must be taken into consideration, as it seems they play a more significant role than natural environmental needs.*

Unfortunately, especially in Europe, there are more examples of the second type as tourism developed quite early, in most cases before formal, legally regulated nature protection. The first scenario was possible only in areas inaccessible due to environmental (e.g. peatbogs or unfavorable relief, as in the case of Biebrza and Narew National Parks in Poland) or political reasons. In the latter case these were usually military training grounds or borderland areas, typical mostly for the so-called Soviet bloc countries in Eastern Europe. The Šumava National Park was established on areas formerly inaccessible due to both political reasons presented above.

Crucial for the research on the ways of tourism management in protected areas is to develop of a classification of such spaces not so much in terms of the protection regime, but precisely taking into account ways they are made accessible for tourists. There are different types of protected areas in individual countries, and even within one country the same legal form of nature protection may be associated with different models of tourism management.

## Acknowledgments

This work was supported by the University of Wroclaw under grant no. 0420/2667/18.

**Open Access** This article is distributed under the terms of the Creative Commons Attribution 4.0 International License (http://creativecommons.org/licenses/by/4.0/), which permits unrestricted use, distribution, and reproduction in any medium, provided you give appropriate credit to the original author(s) and the source, provide a link to the Creative Commons license, and indicate if changes were made.
